# Type 2 Diabetes: Insulin Resistance May Be the Result of Mitochondrial Dysfunction

**DOI:** 10.1371/journal.pmed.0020292

**Published:** 2005-08-16

The role of insulin resistance (IR) in type 2 diabetes, the most frequently encountered metabolic disorder in the world, has attracted much attention in recent years. Virtually all patients with type 2 diabetes have IR, which usually appears some 10–20 years before the disease itself. Although the existence of the relationship between IR and type 2 diabetes is well recognized, the underlying mechanisms are poorly understood. A study by Kitt Falk Petersen and colleagues provides important new information on the underlying pathogenic mechanisms that lead to the development of IR.

Recent findings have suggested that inherited defects in mitochondrial oxidative phosphorylation activity might play a key role in the development of IR. Studies have demonstrated a relationship between dysregulated fatty acid metabolism, fat accumulation in muscle cells, and IR in skeletal muscle. This fat accumulation appears to interfere with insulin signaling, resulting in reduced insulin-stimulated muscle glucose transport activity and decreased muscle glycogen synthesis. Magnetic resonance spectroscopy (MRS) studies have found reduced basal rates of muscle mitochondrial ATP production, associated with increased intramyocellular lipid content. Consistent with these results, microarray studies have found a coordinated reduction in PGC-1á-responsive genes in patients with obesity and type 2 diabetes and their overweight first-degree relatives.

Building on these findings, Petersen and colleagues examined insulin-stimulated rates of muscle ATP synthesis and phosphate transport, to investigate whether mitochondrial function is affected not only in the fasting state but also during insulin stimulation and to determine whether inherited defects in mitochondrial oxidative phosphorylation activity might be responsible for IR.

Participants in the study were in their late 20s, lean, nonsmoking individuals with IR who were offspring of parents with type 2 diabetes. The participants were thus selected to be free of other risk factors for IR such as obesity and smoking. Such individuals are ideal for examining the earliest metabolic defects responsible for the pathogenesis of IR since they have none of the confounding factors that are likely to be present in patients with type 2 diabetes. A metabolic defect in this group is likely to be an early event of genetic origin and, therefore, is potentially a primary cause of the later development of diabetes. The individuals in the control group were healthy, nonsmoking, and matched for age and weight to the individuals in the IR group. Insulin was administered using a hyperinsulinemic-euglycemic clamp, and rates of mitochondrial phosphorylation activity (muscle ATP synthesis) were assessed by MRS. [Fig pmed-0020292-g001]

**Figure pmed-0020292-g001:**
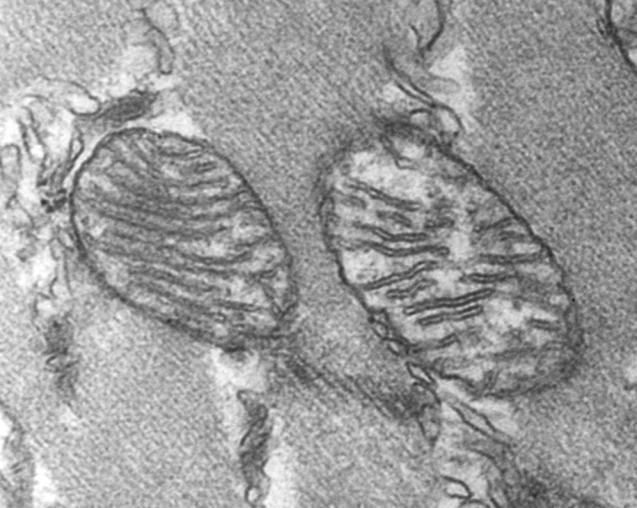
Mitochondria in human muscle cell

Rates of insulin-stimulated glucose uptake were decreased by approximately 50% in the individuals with IR compared to the controls and were associated with an approximately 2-fold increase in intramyocellular lipid content. In the control individuals, rates of ATP synthesis increased by approximately 90% during the hyperinsulinemic-euglycemic clamp. In contrast, insulin-stimulated rates of muscle mitochondrial ATP synthesis increased by only 5% in the individuals with IR. This small increase in muscle mitochondrial ATP synthesis in the individuals with IR was associated with a severe reduction of insulin-stimulated increases in intramyocellular phosphorus concentrations.

The authors say their study provides further evidence that IR in skeletal muscle of individuals with IR who are offspring of parents with type 2 diabetes may be related to defects in mitochondrial dysfunction. Furthermore, there were also severe defects in insulin-stimulated phosphate transport into skeletal muscle in the individuals with IR, which may be part of the defect leading to impaired ATP synthesis in the muscle of these individuals.

The implications of the study are also discussed in a Perspective by Anton Wagenmakers (DOI: 10.1371/journal.pmed.0020289). Although he suggests that the defects underlying IR are likely also to involve organs other than muscle, he notes the clinical relevance of the main finding, which might explain the weight maintenance problems that individuals with obesity and IR have.

